# Insights into the Regulatory Roles of miRNAs in the Salivary Glands of the Soft Ticks *Ornithodoros moubata* and *Ornithodoros erraticus*

**DOI:** 10.3390/pathogens14060595

**Published:** 2025-06-17

**Authors:** Ana Laura Cano-Argüelles, Ricardo Pérez-Sánchez, Cristian Gallardo-Escárate, Rocío Vizcaíno-Marín, María González-Sánchez, Ana Oleaga

**Affiliations:** 1 Parasitología Animal, Instituto de Recursos Naturales y Agrobiología de Salamanca, (IRNASA-CSIC), Cordel de Merinas, 40-52, 37008 Salamanca, Spain; ricardo.perez@irnasa.csic.es (R.P.-S.); rocio.vizcaino@irnasa.csic.es (R.V.-M.);; 2 Laboratory of Biotechnology and Aquatic Genomics, Department of Oceanography, University of Concepcion, Concepcion 4030000, Chile; crisgallardo@oceanografia.udec.cl

**Keywords:** *Ornithodoros moubata*, Ornithodoros erraticus, microRNA, antagomir, salivary glands

## Abstract

MicroRNAs (miRNAs) are small non-coding RNAs that regulate gene expression by inhibiting or degrading messenger RNAs (mRNAs). In ticks, salivary miRNAs are proposed to play key roles in modulating host–vector interactions during blood feeding. Previously, we identified salivary miRNAs in *Ornithodoros moubata* and *Ornithodoros erraticus*, major vectors of African swine fever and tick-borne human relapsing fever. In this study, we investigated the regulatory roles of salivary miRNAs in tick biology. Salivary miRNA datasets were re-analysed to identify conserved miRNAs, and putative target genes were predicted using the sialotranscriptomes of both species. In silico predictions were validated through experimental inhibition of specific miRNAs using antagomirs. Knockdown of miR-375 and miR-1 significantly reduced blood intake, oviposition, and fertility, indicating their involvement in feeding and reproductive processes. Silencing miR-252b in *O. moubata* led to increased mortality, suggesting a critical role in survival. Notably, Metis1 was identified as a likely target of miR-252b, and its dysregulation may underlie the observed lethality in miR-252b-silenced ticks. These findings highlight the functional relevance of salivary miRNAs in tick physiology and host interaction, offering new perspectives for the development of innovative tick control strategies.

## 1. Introduction

Ticks are haematophagous arthropods belonging to two principal families, Ixodidae (hard ticks) and Argasidae (soft ticks), which exhibit marked morphological and biological differences. Both families are of considerable medical and veterinary importance, as they serve as vectors for a wide array of pathogens—including viruses, bacteria, protozoa, and helminths—that cause serious diseases in livestock, companion animals, and humans [1]. Climate change is contributing both to the geographical expansion of known tick-borne diseases and to the emergence of novel tick-borne pathogens, prompting international concern [2,3]. In addition, tick feeding may cause direct harm, such as irritation, anaemia, tissue destruction, paralysis, toxicosis, and allergic reactions. Of particular note is Alpha-Gal Syndrome, an allergic condition triggered by the galactose-α-1,3-galactose (α-Gal) epitope found in tick salivary proteins, the incidence of which has markedly increased over the past decade [4,5].

In order to successfully obtain a blood meal, ticks must overcome the host’s haemostatic, inflammatory, and immune defences. To this end, ticks have evolved a complex salivary secretion characterised by multifunctionality, redundancy, and adaptability, reflecting both the complexity and redundancy of host defences and the diversity of vertebrate hosts [6,7,8]. Tick saliva comprises hundreds of proteins as well as non-proteinaceous components, including lipid derivatives and non-coding RNAs (ncRNAs) such as microRNAs (miRNAs) [9,10,11].

miRNAs represent a significant class of regulatory small RNAs (sRNAs) that govern gene expression at the post-transcriptional level. miRNAs can suppress protein translation or induce degradation of target messenger RNAs (mRNAs) by binding to complementary sequences, primarily within the 3′ untranslated region (UTR), but also within the 5′-UTR and coding sequences [12]. Thus, the function of a given miRNA is intrinsically linked to the biological roles of its target mRNA(s). The biogenesis of miRNAs is a tightly regulated process. In the canonical pathway, transcription of specific genes by RNA polymerase II or III produces primary miRNA transcripts (pri-miRNAs) with characteristic hairpin structures. These are processed in the nucleus by the Drosha-DGCR8 (DiGeorge syndrome critical region 8) complex to yield precursor miRNAs (pre-miRNAs), which are exported to the cytoplasm via Exportin-5. In the cytoplasm, the Dicer endonuclease cleaves the pre-miRNA to generate a 20–22 nucleotide double-stranded miRNA. One strand, termed the guide strand, is incorporated into the RNA-induced knockdown complex (RISC), while the complementary (passenger) strand is typically degraded. The mature miRNA within RISC serves as a guide, directing the complex to the corresponding target mRNA [13].

To date, most research on tick miRNAs has focused on a limited number of ixodid species. These studies have addressed miRNA identification, their expression profiles across developmental stages, tissues, and physiological states, as well as their putative roles in tick biology [14,15,16,17] and in the modulation of the tick–pathogen interface [18,19,20]. Findings to date suggest that miRNAs can enhance pathogen transmission and modulate essential biological processes, including oviposition, blood digestion, and moulting. Moreover, miRNAs present in tick saliva are increasingly recognised as key players in tick–host interactions, where they influence gene expression in the vertebrate host at the bite site [10,11,21].

In contrast, research into miRNAs in argasid ticks remains limited. To date, only one study has investigated small RNAs, including miRNAs, in the saliva of two argasid species, *Ornithodoros moubata* and *Ornithodoros erraticus*, the principal vectors of African swine fever (ASF) virus and tick-borne relapsing fever (TBRF) spirochetes in the Mediterranean Basin and south-eastern Africa, respectively [22,23,24,25]. Cano-Argüelles et al. [10] identified 72 conserved miRNA families and conducted in silico predictions and functional analyses of their putative target genes in the swine host. The results suggest their involvement in key biological processes relevant to tick–host interactions, including the regulation of mRNA transcription and gene expression, synaptic activity, immune responses, angiogenesis, and vascular development in the host. However, no information is currently available regarding the role of salivary miRNAs in the physiology of *Ornithodoros* ticks.

A more comprehensive understanding of the regulation of essential genes involved in key physiological processes within the salivary glands of *O. moubata* and *O. erraticus* may inform the identification of more effective antigens for anti-tick vaccine development. Accordingly, the present study aims to investigate the role of miRNAs in the regulation of biological processes in the salivary glands of these two *Ornithodoros* species. To this end, the salivary miRNA dataset obtained by Cano-Argüelles et al. [10] was re-analysed to identify conserved miRNAs, and their putative target genes within the salivary glands were predicted using the sialotranscriptomes of *O. moubata* and *O. erraticus* reported by Oleaga et al. [26] and Pérez-Sánchez et al. [27]. Finally, the in silico predictions were experimentally validated through a miRNA inhibition approach using antagomirs.

## 2. Materials and Methods

### 2.1. Small RNA Sequences Dataset

For the current study, the starting materials were the datasets of small RNAs obtained from the saliva of *O. moubata* and *O. erraticus*, as reported by Cano-Argüelles et al. [10]. Briefly, triplicate samples of saliva were collected from unfed female specimens from both species, and the small RNA fraction was subsequently purified and sequenced using Illumina technology (Illumina NovaSeq 6000, Genomics Services of the Fundación Parque Científico de Madrid, Spain). The generated raw data were deposited in the National Centre for Biotechnology Information (NCBI) (https://www.ncbi.nlm.nih.gov/, accessed on 28 March 2023) under the BioProject accession code PRJNA931918 and Sequence Read Archive (SRA) accession numbers SRR23347806, SRR23347807, and SRR23347808 from *O. moubata* and PRJNA666995, SRR23347809, SRR23347810, and SRR23347811 from *O. erraticus*. Processed reads from each sample were mapped to a non-coding RNA sequence (ncRNA) database in RNAcentral (https://rnacentral.org/, accessed on 5 May 2022). The aim of the study by Cano-Argüelles [10] was to identify salivary miRNAs and other sRNAs and to investigate their regulation of target genes in the host *Sus scrofa*. In the present study, we re-analysed the data using a pipeline designed to identify mature and conserved miRNAs in order to predict their potential mRNA targets in the salivary glands and to provide insight into the role of these molecules in the biological processes within this tissue.

### 2.2. New Bioinformatics Analysis of the Salivary miRNA

Raw sequences were re-analysed using the RNA-seq and miRNA analysis modules in CLC Genomics Workbench version 23.0.2. (Qiagen, Hilden, Germany, https://digitalinsights.qiagen.com). During this process, Illumina raw reads with a PHRED quality score below 24, together with reads containing two or more ambiguous nucleotides (nt), were discarded, and adaptor sequences were trimmed. Subsequently, mature microRNAs were identified as follows. Reads ranging from 18 to 55 nt in length were mapped using the miRBase database, version 22 (www.mirbase.org, accessed on 28 March 2023) [28], allowing for up to two mismatches. Mapping was performed against five arthropod species selected based on their phylogenetic proximity to *Ornithodoros* sp. (namely, *Ixodes scapularis*, *Rhipicephalus microplus*, *Parasteatoda tepidariorum*, *Tetranychus urticae*, and *Drosophila melanogaster*) and additionally against *Homo sapiens*, which was included as a reference model organism. The parameters within the CLC Genomics Workbench were set as follows: minimum length fraction and minimum similarity length fraction of 0.8, mismatch cost of 2, and insertion and deletion costs of 3. Expression levels were quantified as transcripts per million (TPM), and only sequences with TPM values greater than zero across all three biological replicates were retained for downstream analysis.

### 2.3. Validation of miRNA Expression in Salivary Glands by Quantitative RT-PCR

To confirm the presence of miRNAs previously identified in the saliva of *O. erraticus* and *O. moubata* within their salivary gland tissues, four miRNAs shared by both species were selected for amplification by quantitative RT-PCR (qPCR): miR-252b, miR-375, miR-279, and miR-1.

For this assay, three batches of salivary glands (10 pairs per batch) of each species were dissected from unfed female ticks following the procedure described by Oleaga et al. [21]. Briefly, prior to dissection, ticks were subjected to a series of washes comprising tap water, 3% hydrogen peroxide, two rinses in distilled water, 70% ethanol, and a final two rinses in distilled water. Dissections were carried out in ice-cold phosphate buffered saline (PBS), pH 7.4, treated with 0.1% diethyl pyrocarbonate (DEPC), and the salivary glands were excised and immediately preserved in RNAlater solution (Sigma-Aldrich, St. Louis, MO, USA).

miRNAs were purified from the salivary gland tissue samples using the miRNeasy Tissue/Cells Advanced Micro Kit (Qiagen, Hilden, Germany), following the manufacturer’s instructions. The miRNAs were quantified using a fluorometric assay with the Qubit™ microRNA Assay Kit and the Qubit 4 fluorimeter (Invitrogen, Waltham, MA, USA). 

After that, cDNA was synthesised from 200 ng of miRNAs per replicate using the MirX™ miRNA FirstStrand Synthesis kit (Takara Bio, Shiga, Japan), following the manufacturer’s protocol. Amplification was performed in 96-well plates using TB Green^®^ qRTPCR (Takara Bio, Shiga, Japan) under the following cycling conditions: denaturalisation for 10 s at 95 °C followed by 40 cycles of 5 s at 95 °C and 20 s at 60 °C. Each biological sample was amplified in triplicate (2 µL cDNA per replicate). The miRNA-specific forward primers are listed in Table S1 and were used in conjunction with the universal reverse primer provided in the MirX™ miRNA First-Strand Synthesis kit (Takara Bio, Shiga, Japan). Amplification was performed using the ABI PRISM 7900HT Fast Real-Time PCR System (Applied Biosystems, MA, USA). 

Amplification curves were generated using the mean signal intensity values (ΔRn) obtained from the technical replicates for each biological replicate (three salivary gland samples per species analysed) and were visualised using GraphPad Prism version 10.2.1 (GraphPad Software, Waltham, MA, USA).

### 2.4. Computational Prediction of miRNA Targets from Salivary Gland Transcriptome Data

miRNAs regulate gene expression primarily by binding to the 3’ UTR of their target mRNAs, thereby exerting a negative influence on gene expression [29]. Currently, the salivary gland transcriptomes of *O. moubata* and *O. erraticus* lack annotated 3′ UTRs, and no reference genome is available for either species. Consequently, 3′ UTR prediction was first carried out using the ExUTR tool [30], which facilitates the identification of these sequences in non-model organisms from existing next-generation sequencing (NGS) data. 

Briefly, the software workflow was as follows: transcripts were subjected to open reading frame (ORF) prediction followed by ORF validation through alignment with the UniProt database (release 2023_05, https://www.uniprot.org/, accessed on 9 August 2023) using BLASTP. Validated ORFs were subsequently aligned against their corresponding transcript sequences via self-BLAST to identify stop codons. The 3’ UTR sequences were then extracted following the protocol described by Huang and Teeling [30]. 

The 3’ UTR prediction was performed using the consensus salivary gland transcriptomes of *O. moubata* (BioProject PRJNA666995) [26] and *O. erraticus* (BioProject PRJNA931918) [27]. Only transcripts containing a complete ORF and exhibiting a reads per kilobase million (RPKM) value > 1 were selected for 3′ UTR prediction using the ExUTR tool [30].

Next, miRNA target gene prediction was performed using mature miRNA annotated in miRBase and the predicted 3′UTR sequences from salivary gland transcriptomes. This analysis was conducted using the miRNAconsTarget tool from the sRNAtoolbox [31], which employs four programs: simple seed analysis, miRanda [32], TargetSpy [33], and PITA [34]. To reduce false positive target predictions, only salivary transcripts detected by all four programmes were considered. 

### 2.5. Enrichment Analysis of Biological Processes of mRNA Target Predicted

Gene Ontology (GO) term enrichment analysis was conducted to identify biological processes associated with predicting miRNA target genes.

Differential gene expression analyses of the salivary glands of *O. erraticus* and *O. moubata* throughout their trophogonic cycle revealed that the most substantial gene up- and down-regulation occurred seven days post-feeding and identified the genes involved [26,27]. Based on these findings, the present study conducted GO biological process enrichment analysis on the predicted miRNA target genes that were either up-regulated or down-regulated at seven days after feeding.

The analysis was performed using TBtools [35] and the GO database (version 1.2, 01/04/2023 release; https://doi.org/10.5281/zenodo.7796232, accessed 30 April 2023). Only biological processes with a *p*-value < 0.05 were considered significantly enriched.

### 2.6. Effect of miRNA Knockdown on O. moubata Biology

To further investigate the role of miRNAs in the biology of soft ticks, a knockdown experiment with antagomirs was conducted in *O. moubata*, targeting three miRNAs identified in its salivary glands. 

Antagomirs are chemically modified single-stranded miRNA-specific antisense oligonucleotides that are used to bind mature miRNAs and block their activity [36,37]. These modifications consist of (i) 2′-methoxy throughout the entire antisense strand, (ii) 2 phosphorothioates at the 5′ end, and (iii) 4 phosphorothioates plus 4 cholesterol moieties at the 3′ end. 

The miRNA knockdown was induced in three separate batches of *O. moubata* females by administering specific antagomirs targeting miR-252b (Ant-miR252b), miR-375 (Ant-miR375), and miR-1 (Ant-miR1). Specific antagomirs and a non-related antagomir known as antagomir negative control (Ant-NC) (MNH00000) were purchased from Applied Biological Materials Inc. (abm, BC, Canada). Their sequences are shown in Table S2.

The efficiency of miRNA knockdown was assessed by quantifying miRNA levels using qPCR. To evaluate the phenotypic effects, antagomir-treated *O. moubata* females were allowed to feed on rabbits.

#### 2.6.1. Dynamics of miRNA Knockdown in *O. moubata* Females

Prior to undertaking the miRNA knockdown experiment, an in vivo assay was conducted to evaluate the extent and duration of miR-252b knockdown in the salivary glands following administration of its specific antagomir (Ant-miR252b). This preliminary assay aimed to determine the optimal time point for assessing the phenotypic effects of miRNA knockdown in *O. moubata* females. To this end, three post-injection time points were assessed: 24 h, 48 h, and 96 h. 

A total of 75 unfed *O. moubata* females were microinjected in the lower right quadrant of the ventral surface with 0.5 µL of 100 µM Ant-miR252b. In parallel, a control group of the same size was microinjected with 0.5 µL of 100 µM Ant-NC. Prior to antagomir injection, all *O. moubata* females were subjected to a series of washes, as outlined in Section 2.3. Injections were performed using a 5 µL Hamilton syringe fitted with a 33-gauge needle. The syringe was washed up to 15 times with 3% hydrogen peroxide, followed by sterile distilled water prior to use. 

After injection, ticks were kept in a climate-controlled chamber at 28 °C, 85% relative humidity, and a 12 h light/dark cycle. At the designated time points post-injection (24 h, 48 h, and 96 h), two biological replicates, each comprising 10 pairs of salivary glands, were collected from each group (Ant-miR252b and Ant-NC). Dissection of salivary glands, as well as purification and quantification of miRNAs, was conducted as described in Section 2.3. Similarly, cDNA synthesis and qPCR of miR-252b following antagomir-mediated knockdown were performed according to the methodology detailed in Section 2.3. The expression level of miR-252b was normalised to U6 small nuclear RNA (U6 snRNA), a non-coding RNA frequently used as a housekeeping reference gene in small RNA quantification assays, particularly those involving miRNAs [38]. The primers used for U6 amplification are listed in Table S1. Relative expression levels of miR-252b were calculated using the 2^−ΔΔCt^ method, and results are presented as mean ± standard deviation (SD).

#### 2.6.2. Antagomir Treatment in *O. moubata* Females and Assessment of Phenotypic Effects

As outlined in the Results section, our preceding experiment demonstrated that miR-252b knockdown reached approximately 95% at 96 h post-antagomir administration. Based on this outcome, we evaluated the effect of knockdown miR-375, miR-1, and miR-252b on tick biology at 96 h after antagomir injection.

In this assay, four groups of 60 unfed *O. moubata* females were each injected with 0.5 µL of a 100 µM solution of one of the following antagomirs: Ant-miR252b, Ant-miR375, Ant-miR1, or Ant-NC (control). 

Ninety-six hours after antagomir injection, each experimental group was divided into two subgroups: one to evaluate the phenotypic effects of the treatment on tick biology and the other to assess the expression level of miRNA knockdown in the salivary glands of *O. moubata* females.

The first subgroup, comprising three replicates of 15 females from each experimental group (injected with Ant-miR252b, Ant-miR375, Ant-miR1, or Ant-NC) (n = 45), was allowed to feed to repletion on rabbits. Upon detachment, ticks were collected and maintained for 24 h in the climate-controlled chamber at 28 °C, 85% relative humidity, and a 12-h light/dark cycle until coxal fluid emission was complete. Females, which had been weighed prior to feeding, were then weighed again to determine the blood meal amount. Each female was subsequently housed with two males in individual vials and maintained in the climate-controlled chamber for two months to assess survival and reproductive performance, as described by Carnero-Morán et al. [39]. Namely: (i) quantity of blood ingested calculated as the difference in weight before and 24 h after feeding; (ii) female oviposition and fertility rates, namely, number of eggs laid per female and subsequent number of newly hatched nymphs-1 per female; (iii) moulting rate of nymphs-3; and (iv) survival rates of all tested developmental stages at two months after feeding on control and vaccinated rabbits.

The second subgroup, consisting of three replicates of five antagomir-treated females each (N = 15), was used to evaluate the expression levels of the corresponding target miRNA (miR-252b, miR-375, and miR-1). For this purpose, after salivary gland dissection, miRNAs and total RNA fractions were purified using the miRNeasy Tissue/Cells Advanced Micro Kit (Qiagen, Hilden, Germany), as described in Section 2.3. Subsequent cDNA synthesis and qPCR amplification of the target miRNAs followed the procedures outlined in Section 2.3. The expression levels of miR-252b, miR-375, and miR-1 were quantified and normalised against the level of U6 snRNA using the 2^−ΔΔCt^ method, and the results are presented as mean ± SD. The primers used for miRNAs and U6 amplification are listed in Table S1.

### 2.7. Analysis of mRNA Target Genes Following miRNA Knockdown in O. moubata

Six predicted mRNA targets were selected for evaluation of their transcriptional expression levels following miRNA knockdown using qPCR (Table S3).

Total RNA was extracted from *O. moubata* salivary gland samples obtained in the preceding experiment (described in Section 2.6.2) and quantified using a NanoDrop One C spectrophotometer (Thermo Fisher Scientific, San Jose, CA, USA). cDNA synthesis was performed with the RevertAid cDNA Synthesis Kit (Thermo Fisher Scientific, CA, USA) following the manufacturer’s instructions. qPCR reactions were carried out using TB Green Premix Ex Taq II (Tli RNase H Plus) (Takara Bio, Shiga, Japan) under the following thermal cycling conditions: an initial denaturation step at 95 °C for 30 s followed by 40 cycles of 95 °C for 5 s and 59 °C for 34 s.

Expression levels of the selected target genes were quantified and normalised to actin gene expression. Relative mRNA levels were calculated using the 2^−ΔΔCt^ method. The primers used for target mRNAs and actin amplification are listed in Table S1.

### 2.8. Statistical Analysis

To assess the impact of miRNA-specific knockdown on tick biology, the parameters in the groups treated with each antagomir (Ant-miR252b, Ant-miR375, Ant-miR1) were compared with parameters in the control group (Ant-NC) using one-way ANOVA followed by Dunnett’s test. The *p*-values < 0.05 were considered significant. These statistical analyses were conducted using SPSS version 29 software (IBM SPSS Inc., Chicago, IL, USA).

The relative expression of selected miRNAs and target mRNAs after knockdown was compared to the control group (Ant-NC) using an unpaired t-test in GraphPad Prism version 10.2.1 (GraphPad Software, Boston, MA, USA). The *p*-values < 0.05 were considered significant.

## 3. Results

### 3.1. Analysis of Mature Salivary miRNA Identified in miRBase and Validation of Their Expression in Salivary Gland Tissue

Building upon the miRNA NGS data previously reported by Cano-Argüelles et al. [10], this study aimed to improve the identification of salivary miRNAs in female *O. moubata* and *O. erraticus*, with a particular emphasis on evolutionarily conserved mature miRNAs. Additionally, we investigated the potential regulatory roles of salivary miRNAs in biological processes within tick salivary glands. 

miRBase is a widely used repository specialising in miRNA annotation, as it contains sequences from 271 organisms with 48,860 mature miRNAs [28]. The salivary miRNA data from *O. moubata* and *O. erraticus* were mapped to the miRBase and restricted to five species of the phylum Arthropoda (*I. scapularis*, *R. microplus*, *P. tepidariorum*, *T. urticae*, and *D. melanogaster*) and to *H. sapiens*. Up to 91% and 80% of the annotated sequences from *O. moubata* and *O. erraticus* were mapped, respectively, to the tick species *I. scapularis*, and only 0.3% and 0.8% of the sequences from *O. moubata* and *O. erraticus* were mapped, respectively, to *H. sapiens* (Table 1). 

**Figure 1:**
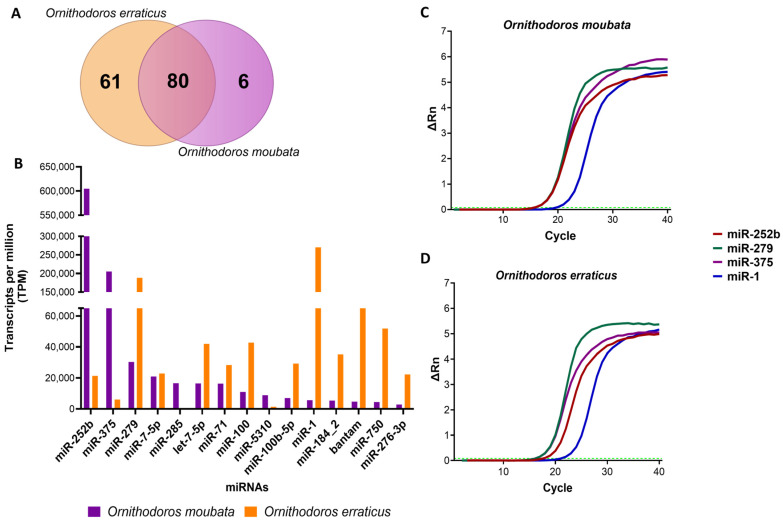
Analysis of mature salivary miRNA identified in *Ornithodoros moubata* and *Ornithodoros erraticus*. (**A**) Number of mature salivary miRNAs annotated in miRBase for both species. (**B**) Fifteen of the most abundant salivary miRNAs in both species expressed as transcripts per million (TPM). (**C**,**D**) qPCR amplification curves from the salivary gland tissue of (**C**) *O. moubata* and (**D**) *O. erraticus*. The green dotted line indicates the C_t_ threshold: 0.208 in *O. moubata* and 0.08 in *O. erraticus*.

The analysis of the raw sequences using the CLC Genomics Workbench software identified 7209 and 16,653 isomiRs in *O. moubata* and *O. erraticus*, respectively (Table S4). These sequences were grouped into consensus sequences, resulting in a final set of 86 mature miRNAs in *O. moubata* and 141 in *O. erraticus*, with 80 shared between both tick species (Figure 1A). Among the most abundant miRNAs in both ticks were miR-252b, miR-375, miR-1, and miR-279, based on their TPM values (Figure 1B, Table S5). The presence of these miRNAs in the salivary gland tissue was confirmed by qPCR in both species (Figure 1C,D).

### 3.2. Prediction of miRNA Target mRNAs from the Salivary Gland Transcriptomes

After confirming the presence of the most abundant salivary miRNAs in the salivary gland tissue of *O. moubata* and *O. erraticus*, the next objective was to identify the target mRNAs potentially regulated by these miRNAs, thereby gaining insight into their functional roles in tick salivary glands. To this end, we conducted in silico target prediction using previously published sialotranscriptomes of *O. moubata* and *O. erraticus* [26,27].

As these sialotranscriptomes lack annotated 3′ UTRs, we first employed the ExtUTR tool to predict 3′ UTRs, considering only transcripts with an ORF longer than 240 nucleotides and expression levels above 1 RPKM (Tables S6 and S7). This resulted in the prediction of 10,788 3′ UTRs from 25,366 transcripts in *O. moubata* and 8487 3′ UTRs from 18,961 transcripts in *O. erraticus* (Table 2; Tables S6 and S7).

**Figure 2:**
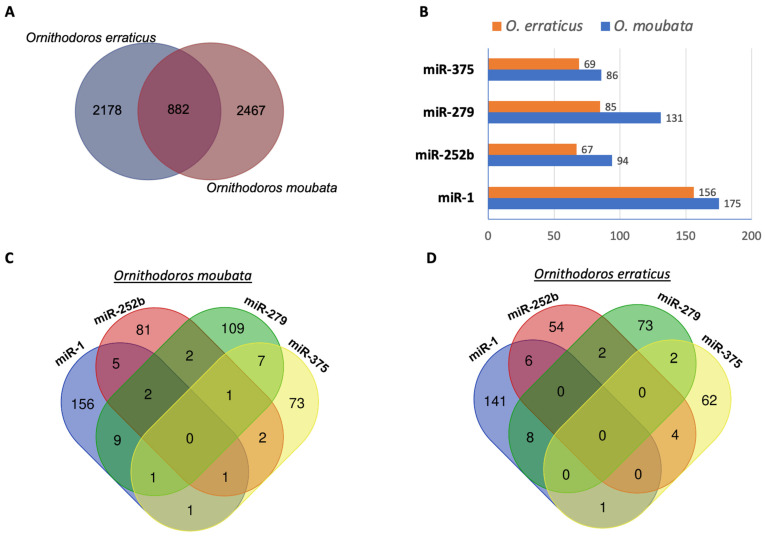
Predicted miRNA target genes in the salivary glands of *Ornithodoros moubata* and *Ornithodoros erraticus*. (**A**) Number of unique and shared target transcripts in both species. (**B**) Number of target transcripts of the most abundant miRNAs (miR-1, miR-252b, miR-279, and miR-375). Number of unique and shared target transcripts for miR-1, miR-252b, miR-279, and miR-375 in (**C**) *O. moubata* and (**D**) *O. erraticus*.

Using these predicted 3′ UTRs, target transcript prediction was carried out for the 86 and 141 mature miRNAs identified in *O. moubata* and *O. erraticus*, respectively (Tables S8 and S9). To minimise false positives, four algorithms were applied (simple seed analysis, miRanda, TargetSpy, and PITA), and only transcripts predicted as targets by all four (referred to as consensus targets) were retained. This analysis identified 11,864 and 13,571 target transcript sequences for *O. moubata* and *O. erraticus*, respectively, corresponding to 3349 and 3060 unique target genes, with 882 shared between the two species (Figure 2A; Table 2; Tables S10 and S11).

Next, given that miR-1, miR-252b, miR-279, and miR-375 are the most abundant salivary miRNAs in *O. moubata* and *O. erraticus*, a detailed analysis of the genes targeted by these miRNAs was performed. In *O. moubata*, 450 unique target transcripts were predicted for these four miRNAs, including 175 for miR-1, 94 for miR-252b, 131 for miR-279, and 86 for miR-375 (Figure 2B, Table S10). Similarly, in *O. erraticus*, 353 unique target transcripts were predicted, including 156 for miR-1, 67 for miR-252b, 85 for miR-279, and 69 for miR-375 (Figure 2B, Table S11). The Venn diagrams in Figure 2C,D show the overlap between the predicted salivary target genes for these four miRNAs in each species. Notably, for each specific miRNA, over 80% of its target genes were unique and not shared with those predicted for the other miRNAs analysed (Figure 2C,D). 

### 3.3. Functional Analysis of Target Genes

After predicting the target genes, we performed an enrichment analysis of the associated biological process. The enrichment analysis was conducted on both up-regulated and down-regulated target genes 7 days after feeding (SG7) compared to their expression level in unfed ticks (SG0). 

**Figure 3:**
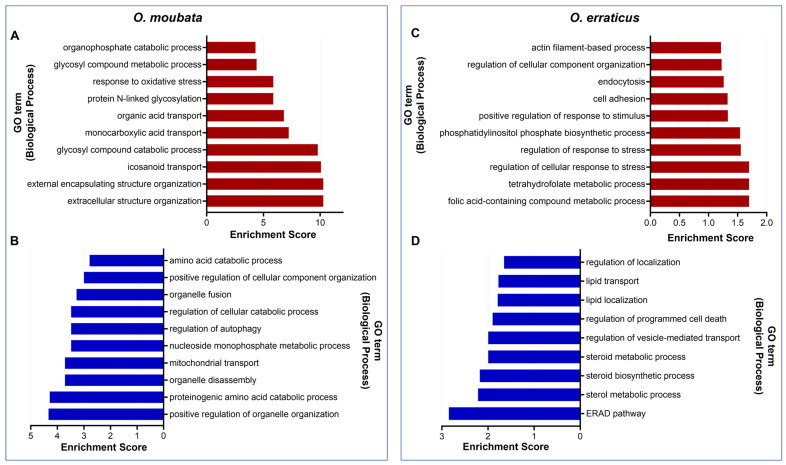
Top 10 enriched biological processes of miRNA target genes up- or down-regulated in female ticks at seven days post-feeding compared to unfed females. (**A**) Up-regulated and (**B**) down-regulated targets in *Ornithodoros moubata*. (**C**) Up-regulated and (**D**) down-regulated targets in *Ornithodoros erraticus*.

This analysis revealed that up-regulated target genes in *O. moubata* were significantly overrepresented (*p* < 0.05) in 46 biological processes, including those related to molecular transport and the metabolism of lipids, carbohydrates, proteins, organic acids, and small molecules (Table S12). Among the biological processes with the highest fold enrichment values, represented in Figure 3A, the most notable are those related to cellular organisation, transport, metabolism, and response to stress. Similarly, down-regulated target genes were significantly enriched (*p* < 0.05) in 35 biological processes, mainly associated with the organisation, biogenesis, and regulation of cellular components, as well as nucleobase, nucleoside, nucleotide, and nucleic acid metabolism (Table S12). The most enriched categories included regulation of cellular processes, such as organelle organisation, autophagy, cellular catabolism, and cellular component organisation (Figure 3B).

In *O. erraticus*, enrichment analysis identified 24 biological processes significantly enriched (*p* < 0.05) among the up-regulated targets, including those related to the organisation of cellular components, regulation of cellular processes, and response to stimuli (Table S13). The biological processes with the highest fold enrichment values were regulating the response to stress, metabolic processes, cell adhesion, and endocytosis (Figure 3C). In addition, nine biological processes were significantly enriched (*p* < 0.05) among down-regulated targets, mainly related to lipid metabolism and transport, regulation of various processes, and stress response, including the ERAD pathway (Figure 3D; Table S13).

### 3.4. miRNA Knockdown Effects on the Biological Function of O. moubata

To investigate the potential regulatory roles of miRNAs in the biology of argasid ticks, *O. moubata* was selected as the model species because *O. moubata* is widely regarded as a reference model for soft ticks due to its well-characterised biology. Moreover, it is more amenable to microinjection techniques and easier to maintain under laboratory conditions than *O. erraticus*.

Therefore, to explore the roles of miR-252b, miR-375, and miR-1 in tick biology, specific antagomirs were injected into female *O. moubata* ticks to inhibit the expression of these miRNAs. Subsequently, miRNA expression levels were assessed, and phenotypic effects in antagomir-treated female ticks were evaluated following blood feeding on rabbits, focusing on feeding, reproductive performance, and mortality.

**Figure 4:**
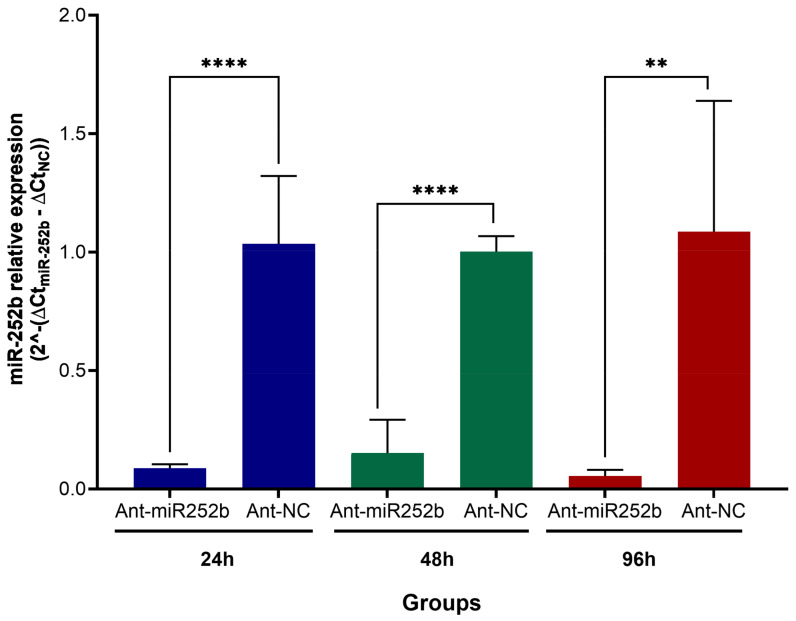
miR-252b expression levels in *Ornithodoros moubata* at 24, 48, and 96 h following the administration of antagomir Ant-miR252b or the nonrelated antagomir Ant-NC used as a negative control. The relative expression level values are presented as the mean ± SD of 2^−ΔΔCt^, calculated by comparing the Ant-miR252b group with the Ant-NC group. Means in ticks treated with antagomir Ant-miR252b were compared to ticks treated with Ant-NC (control) using an unpaired *t*-test (** *p* < 0.01, **** *p* < 0.0001). The human U6 snRNA was used as an internal control.

A preliminary assay was conducted to determine the knockdown kinetics following antagomir administration in order to establish the optimal time point for phenotypic assessment. The expression level of miR-252b—the most abundant miRNA in *O. moubata* saliva—was measured at 24, 48, and 96 h post-injection with either the specific antagomir (Ant-miR252b) or a negative control (Ant-NC). As shown in Figure 4, miR-252b expression was significantly reduced (*p* < 0.05) by over 86% and remained suppressed throughout the experiment compared to the Ant-NC group. At 96 h post-injection, miR-252b knockdown exceeded 95% (Figure 4); therefore, this time point was selected to evaluate the phenotypic effects in the miRNA inhibition assay. 

**Figure 5:**
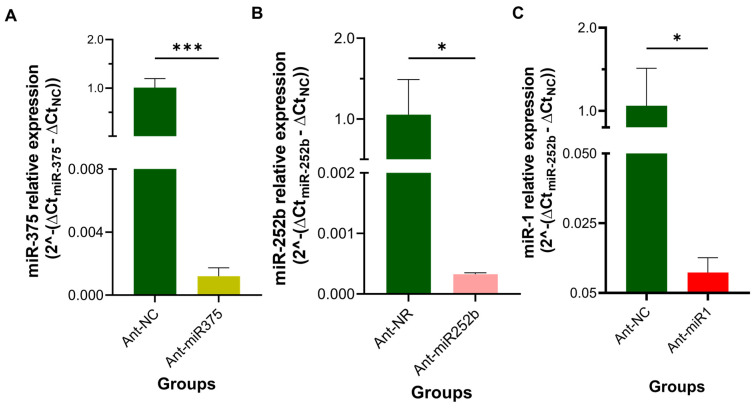
miRNA expression levels in *Ornithodoros moubata* females 96 h after antagomir injection. (**A**) miR-375, (**B**) miR-252b, and (**C**) miR-1 expression levels. The relative expression level values are presented as the mean ± SD of 2^−ΔΔCt^, calculated by comparing each antagomir-treated group (Ant-miR375, Ant-miR252b, Ant-miR1) to the control group (Ant-NC). The human U6 snRNA was used as an internal control. Means in ticks treated with each antagomir were compared to ticks treated with Ant-NC (control) using an unpaired *t*-test (* *p* < 0.05, *** *p* < 0.001).

At 96 h post-injection, a significant knockdown (*p* < 0.05) of miR-375, miR-252b, and miR-1 was observed in females treated with the corresponding antagomirs, with expression levels reduced by nearly 99% compared to the Ant-NC group (Figure 5).

The phenotypic effects of miRNA knockdown in *O. moubata* females are summarised in Table 3. Knockdown of miR-375 and miR-1 led to low but significant reductions in blood intake, oviposition, and fertility (between 11.5% and 15.7%), suggesting a potential role for these miRNAs in regulating feeding and reproductive functions. In contrast, miR-252b knockdown significantly affected survival (6.7% reduction) but had no significant impact on oviposition or fertility. Individual data on oviposition, fertility, survival, and female weight are shown in Table S14.

### 3.5. Quantification of Target Gene Expression Following miRNA Knockdown

Six predicted mRNA targets were selected to assess whether their expression in the salivary glands changed following miRNA knockdown. The selection was made based on the target prediction results presented in Section 3.2, as well as supporting bibliographic evidence. The selected mRNAs were as follows: Metis1 (GIXP02021593), predicted as a target of miR-252b; two Niemann–Pick type C1 domain-containing proteins (NPC1_1 and NPC1_2) (GIXP02047357, GIXP02011494), predicted as targets of miR-375 and miR-1; two ATP-dependent RNA helicases (Hel1 and Hel2) (GIXP02015108, GIXP02053724) identified as targets for miR-375 in mosquitoes [40]; and a heat shock protein 60 (HSP60) (GIXP02024982), identified as a target of miR-1 in *Hyalomma anatolicum* [15]. Further details on these transcripts are provided in Table S3.

**Figure 6:**
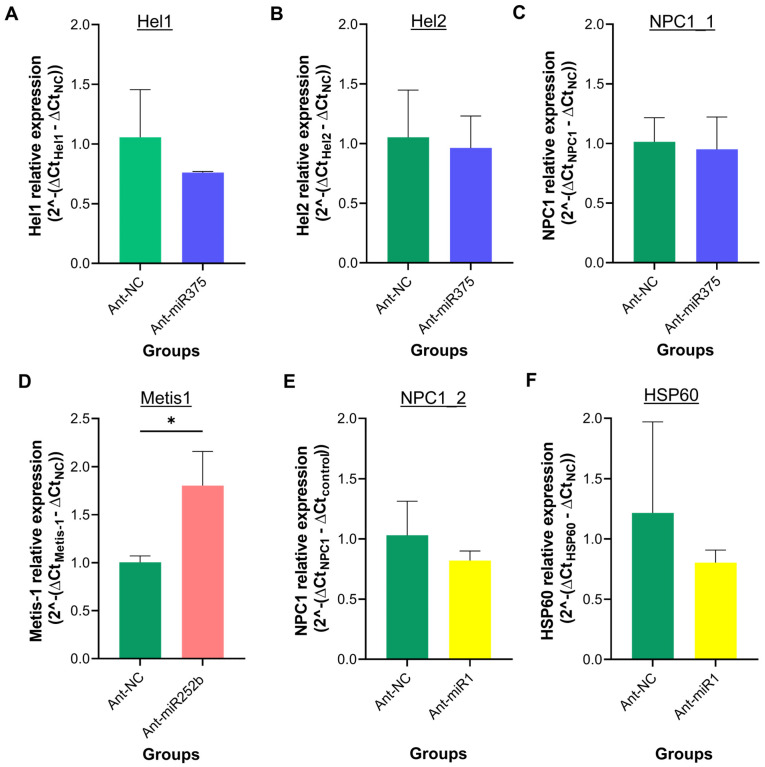
Expression of miRNA target genes in tick salivary glands after miR375, miR-1, and miR-252b knockdown. Relative expression levels of ATP-dependent RNA helicases Hel1 (**A**) and Hel2 (**B**), Niemann–Pick type C1 domain-containing proteins NPC1_1 (**C**) and NPC1_2 (**D**), heat shock protein 60 Hsp60 (**E**), and metalloprotease Metis1 (**F**). Expression levels are presented as the mean ± SD of 2^−ΔΔCt^, calculated by comparing the specific antagomir-treated groups to the Ant-NC-treated group (control). The actin gene was used as an internal control. Means in ticks treated with each antagomir were compared to ticks treated with Ant-NC (control) using an unpaired *t*-test (* *p* < 0.05).

As shown in Figure 6A–C, knockdown of miR-375 resulted in an approximately 20% reduction in Hel1 expression compared to the Ant-NC group; however, this difference was not statistically significant. The expression levels of Hel2 and NPC1_1 remained unchanged. Similarly, following miR-1 knockdown, a slight (~20%) but non-significant decrease in the relative expression of NPC1_2 and HSP60 was observed (*p* > 0.05; Figure 6D, E). In contrast, miR-252b knockdown led to a significant up-regulation of Metis-1 expression (*p* < 0.05; Figure 6F).

These findings suggest that miR-375 and miR-1 may not regulate Hel1, Hel2, NPC1_1, NPC1_2, or HSP60, at least not through mRNA degradation. However, they may exert their regulatory effects via translational repression, which cannot be detected by qPCR. In contrast, Metis1 appears to be a potential transcriptional target of miR-252b, as its expression was significantly up-regulated upon miRNA suppression.

## 4. Discussion

Tick salivary glands are essential for saliva secretion, blood-feeding, and the transmission of tick-borne pathogens [41,42]. Tick saliva is a fluid rich in bioactive molecules—including proteins, lipid derivatives, and ncRNAs, which facilitate the successful acquisition of a blood meal from vertebrate hosts [7] In recent years, increasing attention has been directed towards the role of ncRNAs in tick saliva and salivary glands [43]. Among these, miRNAs—key post-transcriptional regulators of gene expression—have been implicated in tick biology, pathogen transmission, and modulation of the host immune response [11,21,43,44,45]. Despite these insights, the functions and molecular mechanisms of the majority of tick salivary gland miRNAs remain poorly understood. In an effort to address this knowledge gap in argasid ticks, Cano-Argüelles et al. [10] characterised the salivary miRNA content of *O. moubata* and *O. erraticus*. These salivary miRNAs were found to be potentially involved in key biological processes within the host, supporting the hypothesis that miRNAs may play a critical role at the tick–host interface. As indicated, the objective of the present study was to advance our understanding of salivary miRNAs in *O. moubata* and *O. erraticus*, with a particular emphasis on mature miRNAs, and to explore their potential roles in the regulation of tick biology through the prediction of their mRNA targets in the salivary gland transcriptome.

A range of miRNAs are highly conserved throughout evolution, with many shared across species and within specific taxonomic clades, suggesting that these miRNAs may perform fundamental biological functions maintained throughout evolution [46]. Moreover, conservation may facilitate miRNA profiling and identification in newly studied species, as in *R. microplus*, where 87 miRNAs were identified, approximately 70% of which were already known in other species [47].

However, in silico profiling and the discovery of species-specific miRNAs typically require an annotated reference genome. Given the absence of annotated reference genomes for *O. moubata* and *O. erraticus*, the use of reference species for miRNA mapping was essential. In the present study, we identified 86 and 141 conserved salivary miRNAs in *O. moubata* and *O. erraticus*, respectively. Most miRNAs in both argasid ticks were mapped to the hard tick *I. scapularis*, followed by *R. microplus*, *P. tepidariorum*, *T. urticae*, and *D. melanogaster*, as available in miRBase. The predominance of mappings to *I. scapularis* highlights the utility of this species as a reference for soft tick miRNA analysis in the absence of species-specific genomic resources. Moreover, the high degree of overlap in miRNA profiles between *O. moubata* and *O. erraticus* suggests evolutionary conservation within the Argasidae family.

Working with non-model organisms such as *O. moubata* and *O. erraticus* presents several challenges, notably the limited availability of bioinformatics tools optimised for these species. A particular constraint in miRNA research lies in the reliance of most target prediction algorithms on annotated 3′ UTRs, despite increasing evidence that miRNA binding sites are distributed across the entire mRNA sequence, including the 5′ UTR and coding sequence [48]. In the present study, the available sialotranscriptomic data for *O. moubata* and *O. erraticus* lacked 3′ UTR annotation [26,27], thereby limiting the direct application of conventional target prediction approaches. To overcome this limitation, we first implemented the ExUTR pipeline [30] to infer putative 3′ UTR sequences and enable downstream miRNA target prediction. This step was essential for generating a more complete dataset suitable for in silico analysis and underscores the importance of adaptable computational strategies when investigating gene regulation in non-model organisms. 

Although advances in bioinformatics have considerably improved the accuracy of miRNA target prediction, widely used algorithms continue to exhibit relatively high rates of both false negatives and false positives. Computational prediction of miRNA targets remains inherently challenging, as current models often fail to fully capture the complexity of genuine miRNA–target gene interactions. To mitigate these limitations, the use of multiple algorithms based on distinct prediction parameters is generally recommended [49]. The miRNAconsTarget tool, available within the sRNAtoolbox platform, facilitates consensus-based target prediction by integrating four complementary approaches: simple seed analysis, miRanda, PITA, and TargetSpy [50]. The simple seed method identifies potential targets based on the presence of a seed match within a transcript [50]. miRanda assesses sequence complementarity, evolutionary conservation, the thermodynamic stability of the miRNA–mRNA duplex, and the binding site’s position within the 3’ UTR [51]. In contrast, PITA prioritises site accessibility by evaluating the net energetic gain from duplex formation relative to the energy cost required to render the target site accessible to the miRNA [34]. Unlike the aforementioned methods, the TargetSpy algorithm employs machine learning techniques, does not require seed sequence matching, and does not rely on evolutionary conservation, thereby enabling the prediction of non-conserved miRNA target sites [33]. 

To investigate the in vivo role of selected miRNAs in *O. moubata* biology, specific antagomirs targeting miR-375, miR-252b, and miR-1 were injected into female specimens to inhibit their function. These miRNAs have been identified in the salivary glands of several ixodid tick species, such as *Haemaphysalis longicornis, R. microplus, I. scapularis*, and *I. ricinus* [21,44,52,53,54]. Our results showed that miR-375 and miR-1 knockdown caused a significant reduction in the ingested blood, oviposition, and fertility, in line with that reported in some ixodid tick species: miR-375 knockdown in *H. longicornis* ticks resulted in reduced oviposition and egg hatching [54], while miR-1 knockdown in *H. anatolicum* ticks led to prolonged engorgement time and developmental deformities in later stages [16]. Notably, knockdown of miR-252b in *O. moubata* caused a significant increase in mortality (*p* < 0.01). To date, no specific role has been reported for this miRNA in ticks, suggesting a potentially novel and essential function in *O. moubata* biology.

The identification and experimental validation of mRNA targets is essential for elucidating the regulatory roles of miRNAs in cellular processes [55]. However, given that a single miRNA can bind to multiple mRNA targets and conversely that a single mRNA transcript may be regulated by several different miRNAs, the complexity of these interactions presents a considerable challenge. Furthermore, in silico target prediction methods frequently generate a large number of putative targets, making the experimental validation of each candidate gene a labour-intensive and time-consuming endeavour [48]. In light of these limitations, we sought to identify potential target mRNAs that were affected in miRNA-silenced *O. moubata* specimens.

To this end, six potential target genes were selected, and their expression levels in the salivary glands were assessed following miRNA knockdown. For miR-375, two ATP-dependent RNA helicases (Hel1 and Hel2) were selected based on previous findings in mosquitoes, where a DEAD box ATP-dependent RNA helicase was identified as a target for miR-375 [40]. In addition, based on the results of the computational target prediction, two Niemann–Pick type C1 domain-containing proteins (NPC1_1 and NPC1_2) were selected to determine their expression level following miR-375 and miR-1 knockdown. The NPC1 proteins play a key role in cholesterol uptake and intracellular transport and have been proposed as promising targets for anti-*O. moubata* vaccine development [56]. Moreover, although not identified through in silico prediction, HSP60 was included in the analysis as a putative miR-1 target based on its prior identification as a miR-1 target in *H. anatolicum* [16]. This finding highlights HSP60 as a biologically relevant candidate warranting further investigation. 

Our results did not reveal significant differences in the expression levels of Hel1, Hel2, NPC1_1, NPC1_2, or HSP60 following the inhibition of miR-375 and miR-1 (Figure 6). It is possible that these transcripts are not true targets of the silenced miRNAs and may have represented false positives arising from the in silico prediction analysis. 

Alternatively, the post-transcriptional regulatory mechanism of these miRNAs may involve translational repression rather than mRNA degradation, in which case transcript abundance would remain unaffected [46]. Further studies will be required to elucidate the regulatory mechanisms underlying the phenotypic effects observed on oviposition and fertility in *O. moubata* following miR-375 and miR-1 knockdown. 

Metis1 was the only transcript that showed increased expression in the salivary glands following miR-252b knockdown. In ticks, metalloproteases are among the most abundant protein families in the salivary glands [57]. Metis1, identified in the salivary glands of ixodids and argasids, belongs to a family of metalloproteases, which are involved in essential biological processes for tick feeding, including fibrinolysis and wound healing [26,27,58]. Notably, vaccination of rabbits with the recombinant protein interferes with blood meal completion, leading to reduced engorgement weight and egg-laying rate in *I. ricinus* females [58]. Our findings suggest that Metis1 may represent a genuine target of miR-252b, and its dysregulation could compromise survival in miR-252b-silenced *O. moubata* females. Further investigations will be required to confirm this hypothesis, including the validation of the interaction between miR-252b and the predicted target mRNA region using in vitro approaches such as the dual-luciferase reporter assay.

## Figures and Tables

**Table 1:** Annotation of miRNAs from *Ornithodoros moubata* and *Ornithodoros erraticus* saliva using the miRBase database. Species for which annotation was restricted, along with the number of mature miRNA entries available in miRBase for each species as of 7 January 2024 (indicated in brackets).

	*Ornithodoros moubata*		*Ornithodoros erraticus*	
	Read Counts	% of Annotated	Read Counts	% of Annotated
Annotated	477,439	-	1,152,801	-
*Ixodes scapularis* (42)	435,567	91.2	925,853	80.3
*Rhipicephalus microplus* (24)	2	0	0	0
*Parasteatoda tepidariorum* (257)	27,438	5.8	166,779	14.5
*Tetranychus urticae* (92)	11,784	2.5	39,811	3.5
*Drosophila melanogaster* (471)	1221	0.26	11,072	1.0
*Homo sapiens* (2693)	1427	0.3	9286	0.8
Unannotated	33,542,931	-	36,405,630	-

**Table 2:** Prediction of miRNA target mRNAs using the 3’ UTR regions identified in the sialotranscriptomes of *Ornithodoros erraticus* and *Ornithodoros moubata*. Target consensus refers to mRNAs predicted by all four programmes: simple seed analysis, miRanda, TargetSpy, and PITA.

	*Ornithodoros moubata*	*Ornithodoros erraticus*
Number of transcripts *	25,366	18,487
Number of predicted 3’ UTRs	10,788	8487
Target consensus	11,878	13,571
Unique mRNA targets	3901	3633
Unique genes	3349	3060

* Sequences annotated with ORFs > 240 nt and RPKM > 1.

**Table 3:** Phenotypic effects of miRNA knockdown induced by antagomir injection (Ant-NC, Ant-miR252b, Ant-miR375, Ant-miR1) in *Ornithodoros moubata* females fed on rabbits. Results are presented as mean ± SD for each treatment group. Percent reduction relative to the control group is indicated in parentheses. Means were compared to the control group using one-way ANOVA followed by Dunnett’s test. *p* < 0.05 was considered statistically significant (* *p* < 0.05, ** *p* < 0.01).

Parameter	Ant-NC (Control)	Ant-miR252b (% Reduction)	Ant-miR375 (% Reduction)	Ant-miR1 (% Reduction)
Ingested blood (mg)	174.4 ± 7.9	174.3 ± 8.0	154.2 ± 12.4 (11.5) **	151.1 ± 5.2 (13.2) **
Survival (%)	97.8 ± 3.64	91.1 ± 3.3 (6.7) **	100	100
Oviposition (eggs/female)	200.4 ± 8.8	186.0 ± 12.7 (7.1)	170.8 ± 15.4 (15.1) **	170.4 ± 14.6 (15.2) **
Fertility (nymphs/female)	190.6 ± 10.6	172.3 ± 13.1 (9.9)	165.5 ± 15.4 (13.4) *	161.3 ± 15.5 (15.7) *

## Data Availability

The *Ornithodoros moubata* and *Ornithodoros erraticus* salivary microtranscriptomic data are available under Bioproject numbers PRJNA931918 and PRJNA666995.

## Supplementary Materials

The following supporting information can be downloaded at: https://www.mdpi.com/article/10.3390/pathogens14060595/s1, Table S1: Primer names and sequences used for the amplification by quantitative RT-PCR (qPCR) of miRNAs and mRNAs; Table S2: Sequences of miR-252b, miR-375, and miR-1 antagomirs, along with the catalogue number of the antagomir used as a negative control. These compounds were purchased from Applied Biological Materials Inc.; Table S3: Selected targets for in vivo validation in *Ornithodoros moubata* females. For transcript code identification, see Table S6; Table S4: Salivary IsomiRs identified in *Ornithodoros moubata* and *Ornithodoros erraticus* using the CLC Genomics Workbench software; Table S5: Mature miRNAs in *Ornithodoros moubata* and *Ornithodoros erraticus*. TPM, transcripts per million; Table S6: Annotated sialotranscriptome of *Ornithodoros moubata*, including sequences longer than 240 nucleotides and with expression levels exceeding 1 RPKM [26]. 3′ UTR regions predicted using the ExtUTR tool in the annotated sialotranscriptome of *Ornithodoros moubata*; Table S7: Annotated sialotranscriptome of *Ornithodoros erraticus*, including sequences longer than 240 nucleotides and with expression levels exceeding 1 RPKM [27]. 3’ UTR regions predicted using the ExtUTR tool in the annotated sialotranscriptome of *Ornithodoros erraticus*; Table S8: Prediction of *Ornithodoros moubata* target mRNAs by each algorithm: seed2-8, miRanda, TargetSpy, and PITA; Table S9: Prediction of *Ornithodoros erraticus* target mRNAs by each algorithm: seed2-8, miRanda, TargetSpy, and PITA; Table S10: Target consensus of *Ornithodoros moubata* predicted by all four programmes (seed2-8, miRanda, TargetSpy, and PITA); Table S11: Target consensus of *Ornithodoros erraticus* predicted by all four programmes (simple seed analysis, miRanda, TargetSpy, and PITA); Table S12: Enrichment analysis of biological processes (GO terms) for target mRNAs predicted in the transcriptome of *Ornithodoros moubata* up-regulated and down-regulated 7 days after feeding; Table S13: Enrichment analysis of biological processes (GO terms) for target mRNAs predicted in the transcriptome of *Ornithodoros erraticus* up-regulated and down-regulated 7 days after feeding. Table S14: Weight, mortality, oviposition, and fertility data of *Ornithodoros moubata* females treated with antagomirs and fed on rabbits. Ingested blood is calculated as the difference between tick weight before feeding and tick weight 24 h after feeding.
