# Supplementary material for: Insights into the Regulatory Roles of miRNAs in the Salivary Glands of the Soft Ticks Ornithodoros moubata and Ornithodoros erraticus
Source: Pathogens. 2025 Jun 17;14(6):595. doi: 10.3390/pathogens14060595 (PMC12196249; doi:10.3390/pathogens14060595)
Supplement: Supplementary file 1 [file pathogens-14-00595-s001.zip › Table S1.pdf]

Table S1. Primer names and sequences used for the amplification by quantitative RT-PCR (qPCR) of miRNAs and mRNAs.

Name	Primer Sequence (5'- 3')
Mir-252b	TTAAGTAGTAGTGCCGCAGGTA
Mir-279	TGACTAGATCCACACTCATCCA
Mir-375	TTTGTTCGTTCGGCTCGAGTTA
Mir-1	ATGGAATGTAAAGAAGTATGGAGTTC
hU6_Forward	GGAACGATACAGAGAAGATTAGC
hU6_Reverse	TGGAACGCTTCACGAATTTGCG
OmMet_Forward	GTTGAAGATGCTCCGTGGTC
OmMet_Reverse	TGGTTTTCACAGCAGGGAGA
OmHel1_Forward	ACATCCAAGCTGTCAACGTC
OmHel1_Reverse	TCATCTTCGAGTTGGCAATCTG
OmHel2_Forward	TGTTTTGTGCGACCCAATGA
OmHel2_Reverse	GTTGAAGCGGTCCATGATCC
OmNPC1_1_Forward	GGAGATGGCCACTTAGACGA
OmNPC1_1_Reverse	TGGCTGTTCATCCAATCCCT
OmNPC1_2_Forward	AGTACCTCACAATGTGGCCG
OmNPC1_2_Reverse	GGTTCACGAGAGACACTGCA
OmHSP60_Forward	GACGGCCCTTAGTGATTGTG
OmHSP60_Reverse	ATGGTCACTTCTCCCACCTG
Om_Actin_Forward	CTCTTCCAGCCTTCCTTCCT
Om_Actin_Reverse	GTGTTGGCGTACAGGTCCTT
