# Supplementary material for: Insights into the Regulatory Roles of miRNAs in the Salivary Glands of the Soft Ticks Ornithodoros moubata and Ornithodoros erraticus
Source: Pathogens. 2025 Jun 17;14(6):595. doi: 10.3390/pathogens14060595 (PMC12196249; doi:10.3390/pathogens14060595)
Supplement: Supplementary file 1 [file pathogens-14-00595-s001.zip › Table S2.pdf]

Table S2. Sequences of miR-252b, miR-375, and miR-1 antagomirs, along with the catalogue number of the antagomir used as a negative control. These compounds were purchased from Applied Biological Materials Inc.

Name	Sequence/ Cat. Number
Antagomir-miR-252b	5’-UACCUGCGGCACUACUACUUAA-3’
Antagomir-miR-375	5’-UAACUCGAGCCGAACGAACAAA-3’
Antagomir-miR-1	5’-CUCCAUACUUCUUUACAUUCCA-3’
Antagomir-NC	MNH00000
