# Supplementary material for: Insights into the Regulatory Roles of miRNAs in the Salivary Glands of the Soft Ticks Ornithodoros moubata and Ornithodoros erraticus
Source: Pathogens. 2025 Jun 17;14(6):595. doi: 10.3390/pathogens14060595 (PMC12196249; doi:10.3390/pathogens14060595)
Supplement: Supplementary file 1 [file pathogens-14-00595-s001.zip › Table S3.pdf]

Table S3. Selected targets for *in vivo* validation in *Ornithodoros moubata* females. For transcript code identification see Table S6.

miRNA	Target	Nickname	Transcript code	NCBI accession code	Molecular Function
miR-252b	Metis1 protein	Metis1	OM_25458	GIXP02021593.1	GO:0004222: metalloendopeptidase activity GO:0006508: proteolysis; GO:0046872: metal ion binding
miR-375	ATP-dependent RNA helicase me31b	Hel1	OM_17558	GIXP02015108.1	GO:0003729: mRNA binding; GO:0000932: P-body; GO:0005524: ATP binding; GO:0034063: stress granule assembly
	ATP-dependent RNA helicase me31b	Hel2	OM_78396	GIXP02053724.1	GO:0034063: stress granule assembly; GO:0000932: P-body; GO:0003724: RNA helicase activity; GO:0003729: mRNA binding; GO:0005524: ATP binding
	Niemann-Pick type C1 domain-containing protein	NPC1_1	OM_66757	GIXP02047357.1	GO:0005509: calcium ion binding; GO:0005319: lipid transporter activity; GO:0016021: integral component of membrane GO:0042632: cholesterol homeostasis
miR-1	Niemann-Pick type C1 domain-containing protein	NPC1_2	OM_59641	GIXP02011494.1	GO:0005319: lipid transporter activity; GO:0005886: plasma membrane; GO:0015485: cholesterol binding; GO:0042632: cholesterol homeostasis
	Hsp60	Hsp60	OM_30002	GIXP02024982.1	GO:0005524: ATP binding; GO:0042026: protein refolding
